# Development of a Quantitative Preference Instrument for Person-Centered Dementia Care—Stage 2: Insights from a Formative Qualitative Study to Design and Pretest a Dementia-Friendly Analytic Hierarchy Process Survey

**DOI:** 10.3390/ijerph19148554

**Published:** 2022-07-13

**Authors:** Wiebke Mohr, Anika Rädke, Adel Afi, Franka Mühlichen, Moritz Platen, Annelie Scharf, Bernhard Michalowsky, Wolfgang Hoffmann

**Affiliations:** 1 German Center for Neurodegenerative Diseases e.V. (DZNE), Site Rostock/Greifswald, Ellernholzstrasse 1-2, D-17487 Greifswald, Germany; anika.raedke@dzne.de (A.R.); adel.afi@dzne.de (A.A.); franka.muehlichen@dzne.de (F.M.); moritz.platen@dzne.de (M.P.); annelie.scharf@dzne.de (A.S.); bernhard.michalowsky@dzne.de (B.M.); wolfgang.hoffmann@uni-greifswald.de (W.H.); 2 Institute for Community Medicine, Section Epidemiology of Health Care and Community Health, University Medicine Greifswald, Ellernholzstrasse 1-2, D-17487 Greifswald, Germany

**Keywords:** patient preferences, dementia, participatory research, survey, shared decision making, multi-criteria decision analysis, analytic hierarchy process, person-centered care

## Abstract

Person-centered care (PCC) requires knowledge about patient preferences. An analytic hierarchy process (AHP) is one approach to *quantify*, *weigh* and *rank* patient preferences suitable for People living with Dementia (PlwD), due to simple pairwise comparisons of individual criteria from a complex decision problem. The objective of the present study was to design and pretest a dementia-friendly AHP survey. **Methods:** Two expert panels consisting of *n* = 4 Dementia Care Managers and *n* = 4 physicians to ensure content-validity, and “thinking-aloud” interviews with *n* = 11 PlwD and *n* = 3 family caregivers to ensure the face validity of the AHP survey. Following a semi-structured interview guide, PlwD were asked to assess appropriateness and comprehensibility. Data, field notes and partial interview transcripts were analyzed with a constant comparative approach, and feedback was incorporated continuously until PlwD had no further comments or struggles with survey completion. Consistency ratios (CRs) were calculated with Microsoft^®^ Excel and ExpertChoice Comparion^®^. **Results:** Three main categories with sub-categories emerged: (1) *Content:* clear task introduction, (sub)criteria description, criteria homogeneity, (sub)criteria appropriateness, retest questions and sociodemography for heterogeneity; (2) *Format:* survey structure, pairwise comparison sequence, survey length, graphical design (incl. AHP scale), survey procedure explanation, survey assistance and response perspective; and (3) *Layout:* easy wording, short sentences and visual aids. Individual CRs ranged from 0.08 to 0.859, and the consolidated CR was 0.37 (0.038). **Conclusions:** Our formative qualitative study provides initial data for the design of a dementia-friendly AHP survey. Consideration of our findings may contribute to face and content validity in future quantitative preference research in dementia.

## 1. Introduction

Dementia diseases represent a challenge for healthcare systems worldwide [1]. Globally, approximately 55 million people live with a form of dementia, with nearly 10 million new cases diagnosed every year [2]. Alzheimer’s disease (AD) and other dementias are estimated as the fourth leading cause of death in people aged 75+ years according to the *Global Burden of Disease Study 2019* [3]. No curative treatment exists for People living with Dementia and mild cognitive impairment (hereafter referred to as ‘PlwD’). PlwD require a timely differential diagnosis [1,4] and care, which ensures a high quality of life (QoL) [5].

Person-centered care (PCC) is the underlying philosophy of the *Alzheimer’s Associations’ Dementia Care Practice Recommendations* [5] committed to improve the QoL of PlwD and has been included in many national guidelines and dementia plans [6,7,8,9,10,11,12]. PCC challenges the traditional clinician-centered/disease-focused medical model with an alternative model of care customized to each person [13]. This customization requires knowledge about the recipients’ preferences [14,15]. Among PlwD, some data is available about preferences for care [16,17,18], mostly elicited by application of qualitative methods, e.g., Harrison Dening et al. [19], and Likert-type scales, e.g., van Haitsma et al. [20]. These methods fall short to *quantify, weigh* and *rank* patient-relevant elements of care to measure their relative importance and, as such, in identifying the most/least preferred choices. The latter can be assessed with quantitative preference instruments from Multi-Criteria Decision Analysis (MCDA) [21]. An example of the latter is a Discrete Choice Experiment (DCE), such as applied by Groenewoud et al. [22] to address relevant aspects of outpatient care and support services for people with AD, although with patient representatives and not patients themselves. Other MCDA techniques commonly used in health care include best–worse scaling (BWS) [23] and the Analytic Hierarchy Process (AHP) [24]. The AHP has been suggested as feasible for elicitation of patient preferences among people with cognitive impairments due to simple pairwise comparisons with only two individual aspects of a complex decision problem [25].

The AHP, like other MCDA-techniques, involves the development of attribute/criteria-based experimental decision models for subsequent design of a quantitative preference instrument [26,27]. One important aspect of the model’s internal validity is appropriate identification and specification of the included attributes/criteria [24,26,27]. Another important step in development of a quantitative preference instrument is thorough pretesting [28]. This can help to establish content validity by ensuring meaningful and culturally competent language and the understandability of instructions (i.e., comprehension), as well as the layout (e.g., length, complexity and overall experience) [28,29]. Particularly in research with PlwD, consideration of these issues is important to ensure a dementia-friendly design of the quantitative preference instrument [30]. Furthermore, the appropriateness of previously defined (sub)criteria [31] and local translations, as well as the comprehensibility of (sub)criteria within the choice sets have to undergo final pretesting.

To the best of our knowledge, no previous research has reported details about the critical development phase of a quantitative preference instrument in dementia care, i.e., an AHP survey. The aim of our study was to fill this gap with a rigorous process report about comprehension and layout to design a dementia-friendly AHP survey, including an assessment of the appropriateness of previously defined (sub)criteria.

## 2. Materials and Methods

### 2.1. Qualitative Approach

For the report of this pretest study, we followed the guidelines for reporting formative qualitative research proposed by Hollin et al. [28], who, i.a., listed language refinement, task/instrument design and testing as common objectives of formative qualitative research, such as this study, to design a quantitative preference instrument. The pretest interviews followed a cognitive interview approach guided by the think-aloud and paraphrasing technique [32] for individual patient interviews. We conducted two focus group interviews [33] as expert panels with (1) internal, i.e., colleagues of the research team on site, dementia-specific qualified nurses (so-called Dementia Care Managers, DCMs) [34,35,36] and (2) physicians.

### 2.2. Theoretical Framework

The overarching AHP study *‘PreDemCare’* [37,38] adopts a sequential mixed-methods design [39] for final instrument development. Details about the *PreDemCare* study can be found in [37,38], and a process outline is provided by Mohr et al. [31]. Based on findings from a previous systematic review [40], stage 1 of the pre-study, including a small expert panel and qualitative interviews [31], was conducted to identify relevant (sub)criteria of PCC to develop an AHP hierarchy of relevance to (future) decision makers. Six criteria with two sub-criteria emerged from analyses: *social relationships (indirect contact and direct contact), cognitive training (passive or active), organization of care (decentralized structures and no shared decision making* vs. *centralized structures and shared decision making), assistance with daily activities (professional or family member), characteristics of care professionals (empathy, education and work experience) and physical activities (alone or group)*. The current report focuses exclusively on stage 2 of the pre-study in the overarching *PreDemCare* study, i.e., the pretest phase for development of a dementia-friendly AHP survey. The to-be-developed quantitative preference instrument is intended to assess patient preferences and physician judgements for PCC, including an assessment of their congruence. Details of the subsequent main study in the *PreDemCare* study [37,38] lie outside the scope of this report.

### 2.3. Researcher Characteristics and Reflexivity

W.M., a public health scientist with qualitative research experience, conducted the pretest interviews. W.M. was overseen by A.R., a public health scientist with many years of experience in quantitative preference research [24,41]. Study nurses in ongoing clinical trials on site (ClinicalTrials.gov identifiers: NCT04741932, NCT01401582, German Clinical Trials Register Reference No.: DRKS00025074) functioned as gatekeepers to access the PlwD for patient interviews.

### 2.4. Sampling Strategy and Process

DCMs for Expert Panel 1 (EP1) were selected from ongoing clinical trials on site. PlwD for the patient interviews were selected by typical case sampling [42,43], a type of purposive sampling [33], from the clinical trial participant pool on site. The gatekeepers emphasized the independence of the present study. Informal caregivers (CGs) were invited to join. Physicians for Expert Panel 2 (EP2) were recruited via personal contact and friendship networks as recommended by Asch et al. [44] based on different specialty fields important in dementia care.

### 2.5. Sampling Adequacy

For sample size determination in a *formative* qualitative study, in order to develop a quantitative preference instrument, we oriented ourselves in Hollin et al. [28]. The authors underline that the focus should not be the number of subjects, which may be different from *general* qualitative research, but the strategic collection of actionable input for the development process. Hence, a diversity of perspectives is required [28].

In the current pretest study, we addressed the latter requirement by including different stakeholders (DCMs, patients and physicians). Furthermore, the sample size for the patient interviews of *n* = 10 was guided by expected saturation point based on experiences from previous formative qualitative research for the development of quantitative preference instruments [45,46,47,48,49,50], as well as restricted access to PlwD due to the ongoing COVID-19 pandemic. Transmission risk during contact with this vulnerable patient group was minimized to the greatest extent possible. All interviews were conducted in accordance with a strict hygiene protocol developed on site.

### 2.6. Sample

The total sample of interview participants was *n* = 22. Expert panels included (1) *n* = 4 DCMs and (2) *n* = 4 physicians. The final patient sample included *n* = 11 PlwD, three of whom were accompanied by *n* = 3 informal CGs as silent supporters. Eligibility criteria for PlwD included ≥60 years of age, indication of mild cognitive impairment (MCI) or early-to-moderate-stage dementia by diagnosis or cognitive test result (e.g., DemTect < 13 [51] or Mini-Mental State Examination (MMSE) < 27 [52,53]), ability to understand written and oral German and written consent provided by patient/legal guardian [37].

### 2.7. Ethical Review

This pretest study was as part of the overarching preference study *PreDemCare* [38] and approved by the Ethics Committee of the University Medicine Greifswald (Ref.-No.: BB018-21, BB018-21a, BB018-21b).

### 2.8. Data Collection Methods, Sources and Instruments

EP1 was conducted on site. Prior to EP1, the researchers developed a first draft of the AHP survey, which was shared with the DCMs in preparation for discussions. During EP1, the DCMs reviewed and discussed the survey’s content, comprehensibility and layout. EP1 was recorded. Field notes taken directly in the survey formed the main data from EP1. Changes were implemented immediately, and an expert-reviewed survey draft was prepared for the patient interviews.

Individual patient interviews were conducted with PlwD and informal CGs in their homes or in day clinics from July to August 2021. Based on our experiences with previous interviews in pre-study stage 1 [31], we chose an individual interview format with a single interviewer (WM), settings known to the patients and the possibility of inviting an informal CG as a silent supporter to create a comfortable interview situation [54]. Participation in the interview required prior provided informed written consent, which could be provided by the PlwD or a legal guardian. All interviews were recorded. The audio recording was started after both the informed consent procedure and introduction of the participants to ensure privacy. The average interview time was 60 min.

We used a semi-structured interview guide oriented in the one proposed by Danner et al. [55] to ensure an efficient interview structure and give the PlwD the opportunity to freely provide feedback about the presented survey. The survey, developed oriented in a previous AHP survey with an aged population proposed by Danner et al. [55], included three paragraphs: (1) information about the content of the survey; (2) the AHP survey, including pairwise comparisons for the a) criteria and b) sub-criteria; and (3) a self-developed sociodemographic questionnaire for patient characteristics and related potential subgroup analyses. The participants were asked to assess the question formulations for their appropriateness and comprehensibility, as well as to provide information about the underlying motivation determining their answers. We used the AHP judgement scale with verbal explanations of numeric values [56]; an example of a graphical display can be found in Hummel et al. [57].

The main source of data was field notes taken on the paper survey presented to each individual patient. These notes included concrete feedback about wording, format, layout and related comprehensibility. After each pretest interview, the study team met for a short debriefing discussion to decide on changes to be implemented in the survey prior to the next pretest interview. Feedback was incorporated on a continuous basis until the PlwD had no further comments/struggles with completion of the survey.

After *n* = 11 patient interviews, EP2 was conducted by W.M. and A.A. with *n* = 4 specialist physicians via LifeSize [58], a video conference software. W.M. acted as 1st moderator and A.A. as 2nd moderator, taking field notes and keeping track of time. The focus group interview was audio-recorded. The DCM- and patient-reviewed survey was translated to a first draft of the physician’s version of the AHP survey, which included the same pairwise comparisons. In the initially drafted version, physicians were asked “to assume the patient’s perspective”, i.e., to answer the survey as a proxy. The content of the complete survey was similar to that of the patient’s version, likewise ending with a short self-developed sociodemographic survey focused on the physician’s background. During EP2, physicians were asked (1) to review the content of the AHP survey for whether or not it included all relevant aspects of person-centered home care for PlwD from an expert point of view; (2) to assess the comprehensibility and layout of the physician’s version, as well as length of the survey; (3) to express their opinions about the respective point of view to answer the survey, i.e., proxy rating or physician’s judgement; and (4) whether all relevant sociodemographic aspects in the physician’s version had been covered. Furthermore, the physicians were encouraged to raise other topics they considered relevant. After EP2, another two patient pretest interviews were performed for a final review of a few changes based on the expert opinion.

### 2.9. Data Processing and Analysis, Incl. Techniques to Enhance Trustworthiness

Data processing and analysis were conducted according to Coast et al. [59], who recommend iterative constant comparative approaches for analyses during the stage of language refinement (i.e., comprehension of (sub)criteria), as these approaches are more efficient than methods whereby data are collected in advance and analyzed afterwards. Iterative studies yield the ability to adapt questions, formulations, layout, etc., in both a timely and continuous manner based on the findings generated in the study process.

Field notes in the patient survey were analyzed on a continuous basis by W.M. and A.A. and overseen by A.R. as a third reviewer. Additionally, partial transcripts of the patient interviews, likewise reviewed by two reviewers with a third reviewer present in case of disagreement, complemented the field notes respective to themes that appeared important with regard to the comprehension and layout of a dementia-friendly AHP survey, as well as the appropriateness of previously defined (sub)criteria. If names were mentioned during the interview, they were replaced, e.g., with “XXX”, to ensure privacy.

Furthermore, AHP data from the completed PlwD surveys were transferred and analyzed with a Microsoft^®^ Excel-based AHP tool [60] and Expert Choice Comparion^®^ [61], to, i.a., review both individual consistency ratios (CRs) and the consolidated result for PlwD by application of arithmetic (geometric) mean for aggregation of individual priorities (judgements) [62]. For CRs, the literature usually recommends a consistency threshold of 0.1–0.2 [57,63]; however, particular circumstances, such as cognitive capacities of surveyed participants, may warrant the acceptance of a higher value at 0.3 [60,64].

Analysis of patient characteristics was based on the self-developed sociodemographic questions. Severity of cognitive impairment was determined during recruitment based on inclusion criteria, cf. Section 2.6, by the internal study nurses as gatekeepers based on their most recent assessment with a validated instrument (MMSE) [52] in the clinical trial from which patients had been recruited.

With our choice of diverse data collection and analysis methods and the inclusion of a diversity of participants and perspectives, we aimed to ensure data triangulation [33]. For quality control, the final versions of the surveys (patients and physicians) were discussed and agreed upon in a final meeting of the research team.

## 3. Results

PlwD characteristics are depicted in Table 1. The majority of DCMs (75%) were women aged between 31 and 40 years with education as geriatric (50%) or registered nurses (50%). Years of work experience in dementia care ranged from 8 to 30 years. All DCMs had previous experience with the Dementia Care Management-intervention developed on site [34,35,36] as part of clinical trials (ClinicalTrials.gov identifiers: NCT04741932, NCT01401582, NCT03359408; German Clinical Trials Register No: DRKS00013555, DRKS00025074), with the number of PlwD previously under their care ranging from 150 to 300 patients. Among physicians, half (50%) were aged 61–70 and 51–60 years, respectively, with the majority (75%) of male gender and employed. All practiced in an urban area in different settings (local health authority, special service health, individual practice or ambulatory healthcare center). Fields of specialty included psychiatry/psychotherapy, general medicine, anesthesiology, pain therapy and palliative care. One was familiar with PCC, and two knew about *Shared Decision-Making*.

Three main categories with sub-categories emerged from data analysis of field notes and partial transcripts. Categories with respective key quotations from all participants are depicted in Supplementary Materials File S1.

### 3.1. Content

#### 3.1.1. Survey Title Page for PlwD

During the previous qualitative interviews [31], we experienced some PlwD to be nervous, as some expected a test and wanted to “perform well”, cf. Supplementary Materials File S1, row 1, which was confirmed by EP1. Hence, we emphasized with bold and underlined font on the title page that the survey does not include a test but asks about the PlwD’s opinion.

#### 3.1.2. Survey Title Page for Physicians

EP2 emphasized that the title page should state the severity of cognitive impairment to consider during completion of the survey clearly, as care and medical needs may differ, cf. Supplementary Materials File S1, row 2, paragraphs (para.(s)) 1–2.

The initial draft of the physician’s survey asked clinicians to answer the survey from the perspective of their patients, i.e., from a proxy perspective. EP2 revealed concerns about this format and potential risk of bias. The panelists recommended to instead ask for an ‘expert opinion’, i.e., physicians’ judgments with respect to their patients, cf. Supplementary Materials File S1, row 2, para.(s) 3–7. The physician’s AHP survey was adapted accordingly.

#### 3.1.3. Description of (Sub)Criteria for PlwD

During the previous interviews [31], we found that extensive technically descriptive sentences of the (sub)criteria should be avoided and that the abstract (sub)criteria titles should be described by concrete examples the PlwD can relate to, which was confirmed by EP1.

During the first pretest, the assisting interviewer observed the participant to repeatedly read all the (sub)criteria-describing examples, which increased the interview time substantially. Hence, we tested the removal of the examples describing the (sub)criteria from the pairwise comparison questions throughout the complete survey from pretests 2 to 8. Without the concrete examples, the reading time was decreased, but the subsequent participants had difficulty understanding the mere abstract titles of the (sub)criteria. Thus, we decided to return the examples describing the (sub)criteria in pretest 9 and included them in the final version of the survey.

#### 3.1.4. Formerly Merged Criteria Demerged

After the card games during the previous interviews [31], we decided to merge two criteria—*‘Attention & support with worries, feelings and memories’* and *‘Social relationships’*—as during the previous stage, participants had indicated overlapping of these two criteria, cf. Mohr et al. [31]. In the current study, one participant expressed confusion about *‘Attention & support with worries, feelings and memories’*. Therefore, we decided to demerge the formerly merged criteria and remove *‘Attention & support with worries, feelings and memories’* from the survey, cf. Supplementary Materials File S1, row 4, para. 1.

#### 3.1.5. AHP Axiom 2

EP2 expressed concerns about the homogeneity of the criteria, in particular related to criterion (6) ‘*Organization of Health Care*’, cf. Supplementary Materials File S1, row 5, para.(s) 1–5. However, one expert emphasized that the pairwise comparisons of the criteria should be considered from the point of view of the PlwD, cf. Supplementary Materials File S1, row 5, para. 6.

#### 3.1.6. Introduction of Sub-Criteria in the PlwD Version of the AHP Survey

Initially, the criteria-describing examples had been stated under the abstract criteria-titles just above the to-be-introduced sub-criteria, cf. Supplementary Materials File S1, row 6, para. 1. To reduce the amount of words in the introduction of the sub-criteria and to avoid behavior such as that described in Section 3.1.3, we decided to remove the criteria-describing examples from the introduction of the sub-criteria.

#### 3.1.7. Appropriateness of (Sub)Criteria

**Figure 1:**
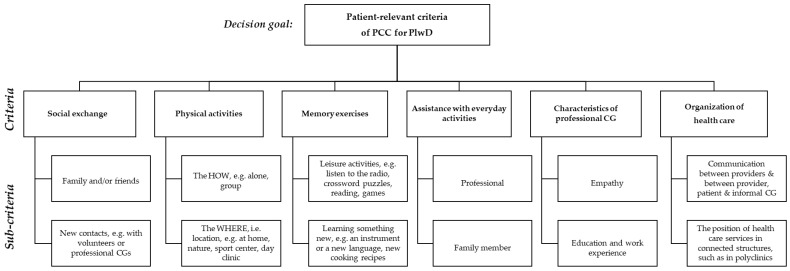
Final AHP hierarchy with patient-relevant (sub)criteria of person-centered dementia care.

EP2 criticized the content of the sub-criteria for (1) *Social Exchange*, (2) *Physical Activity,* (3) *Memory Exercises* and, in particular, (6) *Organization of Health Care*, cf. Supplementary Materials File S1, row 7, para.(s) 1–5. Panelists noted that the respective sub-criteria were presented on an ordinal scale, whereas sub-criteria for criteria 4 and 5 were presented on a nominal scale. Most criticism arose around the sub-criteria for criterion (6), as EP2 did not agree that the structures of health care and shared decision making are correlated, cf. Supplementary Materials File S1, row 7, para.(s) 1–5. W.M., as interviewer, emphasized that the content had been developed based on literature and, first and foremost, on the feedback from the patients, (e.g., Supplementary Materials File S2). We revisited both the transcripts from the previous interviews, as well as field notes and audio recordings from the pretest interviews, to revise the sub-criteria for criteria 1, 2, 3 and 6. The revised sub-criteria were pretested again with two PlwD (pretests 11 and 12), who approved the revised sub-criteria, cf. Supplementary Materials File S1, row 7, para(s). 6–22. Subsequently, the surveys for both populations were finalized, including the changes. The final AHP hierarchy is depicted in Figure 1.

#### 3.1.8. Validity and Inconsistency in the AHP Survey

To check for validity of PlwD responses, we included retest questions. Initially, we had included two example questions. The first example question (*No social relationships* vs. *a lot of social relationships*) asked for a comparison of the extreme ends for criterion (1) *Social Exchange*. The second (*social relationships* vs. *additional cost*) asked for a comparison of the most important and the least important criterion based on the results from the previous qualitative interviews [31]. Additionally, we repeated the pairwise comparison *social exchange* vs. *physical activities* twice. EP1 raised concerns about multiple repetitions of questions, as some patients might be irritated by this, cf. Supplementary Materials File S1, row 8, para.(s) 1–3. Additionally, survey completion during the first patient pretest took more than one hour, cf. Section 3.2.3. Hence, we deleted the second example question and one of the repeated criteria questions to avoid excessive repetitions and to reduce the length of the survey. The sub-criteria comparison for criterion (1) *Social Exchange* was repeated once and was not changed.

The results of retest questions among PlwD are depicted in Table 2. Two participants (pretests 2 and pretest 4) chose differently at the level of criteria, which was also contradictory to their answer in the first example question (no vs. a lot of social exchange). Four participants (pretests 3, 5, 8 and 11) chose the same (sub)criteria but assigned different values, with pretest 8 and 11 showing minor deviations (7 instead of 6 on the rating scale).

The individual CRs among PlwD at the level of criteria are depicted in Table 3a. At the level of sub-criteria, no CRs can be stated, as inconsistency of a pairwise comparison between two (sub)criteria always equals zero. Consolidated CRs for all participants are depicted in Table 3b. Individual CRs ranged from 0.08 to 0.86, with a consolidated CR of *n* = 11 PlwD at 0.37 (0.038) based on the arithmetic (geometric) mean for aggregation of individual priorities (judgements).

#### 3.1.9. Heterogeneity of Respondents

EP2 suggested considering the heterogeneity of the patients and that this may influence the responses, cf. Supplementary Materials File S1, row 9, para. 1. Heterogeneity of respondents and respective potential subgroup analyses in both populations were accounted for by the survey developers through inclusion of a comprehensive self-developed sociodemographic questionnaires. The PlwD sociodemographic questionnaire includes age group, gender, family status, educational status, (previous) occupation, income group, severity of cognitive impairment, regular medication intake, psychosocial treatment(s) and subjective assessment of health status. The physician’s sociodemographic questionnaire includes age group, gender, mode of employment, setting (hospital, individual practice, etc.), area (rural, urban), field of specialty, number of patients with dementia diseases treated currently and in the past, knowledge about PCC and knowledge about shared decision making.

#### 3.1.10. Sociodemographic Questions for PlwD

Both EPs gave positive feedback about our self-developed sociodemographic survey. EP2 suggested including the living situation of PlwD as an additional question for potential subgroup analyses, as preferences may differ, cf. Supplementary Materials File S1, row 10, para.(s) 1–3.

### 3.2. Format

#### 3.2.1. Outline of the Survey

We aimed to accommodate the needs of the patient group in the outline of the final survey, which may differ from those of other AHP surveys. Sociodemographic questions were moved to the end of the survey, as these were deemed easy to complete and would not require much energy, which EP1 agreed upon, cf. Supplementary Materials File S1, row 11, para.(s) 1–2. EP1 suggested to start with the pairwise comparisons at the sub-criteria level, as these comparisons were easier to understand due to the use of icons as visual aids. After discussions among the research team, we decided to start the survey with the most challenging part, the pairwise comparisons at the criteria level, as this part was expected to require the most energy.

#### 3.2.2. Sequence of Criteria-Related Pairwise Comparison Questions

**Figure 2:**
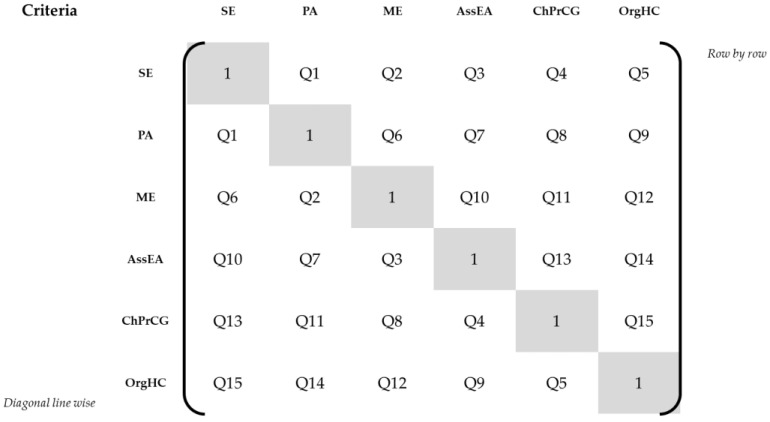
Sequence of criteria pairwise comparisons. Abbreviations: SE = social exchange, PA = physical activities, ME = memory exercises, AssEA = assistance with everyday activities, ChPrCG = characteristics of professional caregivers, OrgHC = organization of health care, Q1–15 = pairwise comparison questions Nos. 1–15.

During pretests 1–4, the sequence of the criteria-related pairwise comparison questions was presented as row-by-row comparisons, as depicted in Figure 2. The first pairwise comparison was *Social Exchange* vs. *Physical Activities*, the second was *Social Exchange* vs. *Memory Exercises*, etc.

Because the participants were confused by repetition of the same criterion in subsequent comparison questions (cf. Supplementary Materials File S1, row 12, para.(s) 1–3), we changed the sequence to diagonal line-wise comparison questions, cf. Figure 2. The first pairwise comparison was *Social Exchange* vs. *Physical Activities*, the second was *Physical Activities* vs. *Memory Exercises*, etc. During pretests 5–8 and 10, we pretested this sequence setup. We observed the highest inconsistency ratio during pretest 6 (85.9%), with a rating of the survey as “rather difficult” by the participant. The participants in pretests 5–8 and 10 continued to criticize the repetition of criteria, despite the better mix of questions. The participants also had a harder time remembering their responses to previous criteria comparison questions, including the same criteria. Hence, the final survey version was changed back to the initial row-by-row sequence of the pairwise comparisons.

#### 3.2.3. Length of Survey

EP1 emphasized that the survey should be kept as short as possible. Despite an effort to shorten the survey substantially, pretest 1 lasted 1 h and 23 min, cf. Supplementary Materials File S1, row 13, para. 1. Subsequently, the content was reduced to the absolute minimum of information that would still allow for enough information to ensure an informed completion of the survey by the participants in the upcoming main study [37].

#### 3.2.4. Formatting

We observed that the survey should be formatted as simply as possible in order to minimize the amount of visual stimuli and thus increase comprehensibility for the PlwD. This includes the choice of an eye-friendly font with of least size 14 for those patients who want to read by themselves. The first PlwD was overwhelmed by the initial page number layout (“page x out of y”). Hence, we changed this to only show “page x”, cf. Supplementary Materials File S1, row 14, para.(s) 1–2.

#### 3.2.5. Layout: Transformation of the AHP Rating Scale

**Figure 3:**
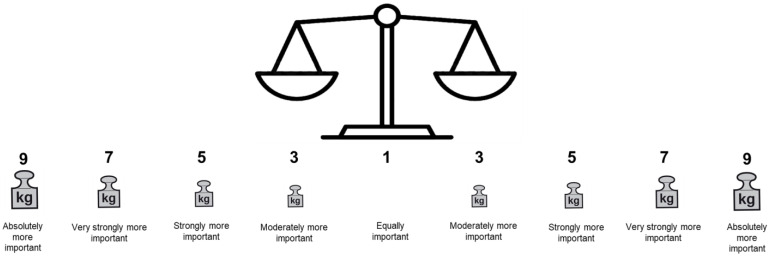
Final layout of the patient-group-adjusted AHP rating scale with verbal judgements [56].

One of the biggest challenges was to adjust the AHP rating scale (layout) to an understandable format for this patient population, which also was emphasized by EP1 with respect to the number of answer options. Our revised AHP rating scale based on participant feedback is depicted in Figure 3.

During pretest 2, W.M. drew a scale similar to that shown in Figure 3 to assist the participant with comprehension of the rating scale. This layout received positive feedback from the participant (cf. Supplementary Materials File S1, row 15, para.(s) 5–9) and was kept for the final version of the AHP survey.

#### 3.2.6. Explanation of Survey Procedure with Pairwise Comparisons

Initially, we used the pairwise comparison of *Social Exchange* vs. *Support with Everyday Activities* as an example to explain the survey procedure, including pairwise comparisons. As the first participant had difficulties with this example (cf. Supplementary Materials File S1, row 16, para. 1), we exchanged it with a simple example involving the choice of a side dish at a restaurant, as described by Danner et al. [55]. This example was received well by the participants (cf. Supplementary Materials File S1, row 16, para.(s) 2–6) and kept for the final survey version.

#### 3.2.7. Simplification of Pairwise Comparisons

During pretest 1, W.M. attempted to assist the PlwD with the pairwise comparisons with “*A or B*” questions (left or right criterion), cf. Supplementary Materials File S1, row 17, para.(s) 1–2. To avoid memory effects, we decided against implementation of “*A or B*” next to the (sub)criteria.

#### 3.2.8. Assistance during Patient Survey

EP1 confirmed our experiences from the previous interviews [31], cf. Supplementary Materials File S1, row 18, para.(s) 1–2. Hence, we decided to have only one and the same interviewer assist with the patient surveys both during the pretest study and planned main study.

#### 3.2.9. Perspective during Responses to Pairwise Comparisons

Some PlwD struggled with which perspective to apply during completion of the AHP survey. The assisting interviewer, W.M., instructed participants to apply today’s perspective and emphasized that this may change in the future, cf. Supplementary Materials File S1, row 19, para.(s) 1–9.

### 3.3. Language

#### 3.3.1. Laypeople Words for “Criteria” and “Sub-Criteria” in a German AHP Survey

For the AHP-related technical terms “criteria” (in German, “Kriterien”) and “sub-criteria” (in German, “Subkriterien”), synonyms understandable by laypeople and, in particular, PlwD had to be identified. “Criteria” were hence termed “characteristics” (in German, “Merkmale”), and sub-criteria, which are usually translated as “manifestations” (in German, “Ausprägungen”) were termed “form of appearance” (in German, “Erscheinungsform”), cf. Supplementary Materials File S1, row 20, para.(s) 1–4.

#### 3.3.2. Avoid Long Sentences

In the survey’s first draft, criteria questions stated: “*In your opinion: Which characteristic of personalized care for the aged living at home do you place greater value on in comparison? And how big is the difference between the two characteristics?*” To shorten questions, this was changed to: “*Which characteristic of care is more important to you and by how much*?”.

During pretest 1, long interrogative clauses halted the progress of the survey, as the participant stopped to reread the questions, despite explanations from W.M. that the question and related task was the same throughout the survey, cf. Supplementary Materials File S1, row 21, para.(s) 1–2. During pretest 2, the participant emphasized difficulties with excessively long sentences, cf. Supplementary Materials File S1, row 21, para.(s) 3–8. Based on these experiences, sentences throughout the survey were shortened substantially. Criteria questions were revised to only state the complete question the first time, whereas subsequent questions stated “*Choice question nr. X*” to avoid experiences as such as those reported during pretest 1.

#### 3.3.3. Choice of Words Matters

Throughout survey development, the choice of words was a topic of concern. Attentiveness to PlwD reactions to wording during the pretest interviews was important, cf. Supplementary Materials File S1, row 22, para.(s) 1–5. EP1 recommended avoiding the use of ‘foreign’ words, e.g., “transparent”.

It was challenging to find titles for the criteria using words that would incorporate the definition of the criteria and, at the same time, be understandable for the PlwD. This was particularly apparent for criterion (1) *Social exchange*, which developed from *“social contact”* to *“social relationships”* to *“social exchange”.* Criterion (1) incorporates *“Provision of different forms of social contact to counterbalance the potentially limited contact with others. This social contact can be real or simulated.”* [40,66]. When asked about social ‘*contact’*, participants tended to broadly relate it to any contact they may encounter, even if not necessarily helpful to counterbalance potentially limited social contact. When asked about social *‘relationships’*, participants tended to relate this to intimate relationships, e.g., marriage, which was too narrow per the definition. Social *‘exchange’* (in German, ‘sozialer Austausch’) was best understood by PlwD to capture the definition of this criterion, cf. Supplementary Materials File S1, row 22, para.(s) 6–12. Similarly, criterion (6) *Organization of health care* was challenging to find a title for that incorporated the definition. Organization of ‘care’ did not incorporate the definition per the participants’ understanding, cf. Supplementary Materials File S1, row 22, para.(s) 15–18. Hence, the title was adjusted to organization of ‘health care’ (in German, ‘Gesundheitswesen’). *‘Cognitive training’*, titled per findings from the previous qualitative interviews, was difficult for participants to understand. *‘Memory exercises’* was better understood, cf. Supplementary Materials File S1, row 22, para. 8.

#### 3.3.4. Use of Icons as Visual Aids

We developed a set of icons as visual aids for sub-criteria for previous qualitative interviews [31]. Due to limited interview time, we were not able to test the appropriateness of these icons during the former interviews. Hence, these were pretested during the pretest interviews and partially adjusted based on patient feedback (cf. Supplementary Materials File S1, row 23, para.(s) 1–12) and EP2 feedback with respect to sub-criteria in general, cf. Section 3.1.7.

## 4. Discussion

Our study provides a rigorous process report about comprehension and layout to design a dementia-friendly AHP survey, including the appropriateness of previously defined (sub)criteria to elicit patient preferences for PCC among community-dwelling PlwD, which, to the best of our knowledge, is the first of its kind. Extensive technically descriptive sentences of the (sub)criteria should be avoided—the abstract (sub)criteria titles should instead be described by concrete examples the PlwD can relate to. The homogeneity of (sub)criteria *(AHP Axiom 2)*, i.e., the comparability of the included elements in the AHP hierarchy, may not always be easy to adhere to when different perspectives need to be accommodated. The appropriateness and the scale at which elements are presented should be reflected upon early by survey developers. As in any AHP study, the validity and consistency of responses may pose a challenge. Layout and presentation of the not-immediately-intuitive AHP scale for an aged and cognitively impaired patient population may be a challenge, but the needs of the population can be accommodated by creativity among survey developers. PlwD may express difficulties with respect to which perspective to apply during completion of an AHP survey; here, the assisting interviewer may help with clarification. The heterogeneity of participants should be considered by including sociodemographic questions that allow for potential subgroup analyses. Assistance is required during completion of an AHP survey with an aged and cognitively impaired patient group.

Extensive technical and abstract description sentences of the (sub)criteria, which are more dependent on cognition, were not observed as helpful for the PlwD to understand the (sub)criteria and their content. As noted in previous qualitative interviews [31], the abstract (sub)criteria became more comprehensible for the PlwD when described by concrete examples the PlwD can relate to. These observations align with previous findings reported by Murdoch et al. [67], who observed greater impairment in those components of language more highly dependent on cognition in people living with AD. Reilly, Troche and Grossman [68] noted sentence comprehension difficulties in AD patients. However, Joubert et al. [69], who studied the comprehension of concrete and abstract words in semantic-variant primary progressive aphasia (svPPA) and AD, found concrete, abstract and abstract emotional words to be processed similarly in the group of AD participants. Patients in the svPPA group were significantly more impaired with respect to processing concrete words than abstract words. Nevertheless, we observed greater difficulties associated with survey completion among our study participants after the descriptive examples had been removed from the pairwise comparisons of the (sub)criteria in order to reduce reading time and the length of the survey. Hence, the final PlwD survey includes concrete descriptive examples for all pairwise comparisons of (sub)criteria, but no technical or abstract descriptive sentences, which are more dependent on cognition.

EP2 expressed concerns about the homogeneity of the criteria. Homogeneity is essential for meaningful comparisons, as the human mind cannot compare widely disparate elements [70]. In the case of considerable disparity between two (sub)criteria, the elements should be placed in separate clusters of comparable size or at different levels altogether [70]. Two physicians noted that pairwise comparisons in particular, including criterion (6) ‘*Organization of Health Care’*, were perceived as incomparable with other criteria, such as (3) *‘Memory Exercises’*. Another physician suggested viewing the pairwise comparisons from the point of view of the PlwD. As mentioned by I1 (W.M.) during EP2 (cf. Supplementary Materials File S1, row 5, para. 2), similar discussions had already occurred in the researcher team. A.R. and W.M. referred to *Axiom 4 (the Axiom of Expectations)*, according which all alternatives, criteria and expectations can be and should be represented in a hierarchy, i.e., that the beliefs of thoughtful individuals should be adequately represented in the decision model [70,71]. Criterion (6) *‘Organization of Health Care’* was observed as the third most important criterion during previous qualitative interviews with PlwD [31]. Based on our previous observations, supported by the argument from one physician and the referral to Axiom 4, we decided to keep Criterion (6) *‘Organization of Health Care’* as part of the AHP hierarchy and survey.

Experts from EP2 criticized the sub-criteria with regard to the different scales these were presented on (ordinal vs. nominal). In particular, the sub-criteria for criteria 1–3 and 6, previously presented on an ordinal scale [31], were criticized for displaying a range from “bad to good”, which was perceived as “tendentious”. The sub-criteria should rather be presented independently on a nominal scale. The latter presentation of sub-criteria corresponds to the suggestion by Hummel, Bridges and IJzerman [57], who presented the sub-criteria independently on a nominal scale for their AHP analysis of the benefits and risks of tissue regeneration to repair small cartilage lesions in the knee. Danner et al. [55], however, presented the sub-criteria for their AHP decision model of treatment characteristics of different treatments for age-related macular degeneration levelled on an ordinal scale. The AHP model for a pilot project to elicit patient preferences in the indication area “depression” by the *Institute for Quality and Efficiency in Germany* [72] presented sub-criteria both on an ordinal and a nominal scale. Particular criticism of the sub-criteria of criterion (6) was shared among the research team, and we decided to revisit the transcripts of previous qualitative interviews, as well as field notes and audio recordings from the pretest interviews to revise sub-criteria for criteria 1, 2, 3 and 6. The revisions were approved by two PlwD during pretests 11 and 12, and the changes were implemented in survey versions for both PlwD and physicians.

Despite the small sample size during this pretest study (*n* = 11 PlwD) we made an initial assessment of validity and internal consistency. As noted by Ozdemir [73], redundancy is required for validity; on the other hand, a small number of comparisons is required for consistency. For the sake of efficiency, AHP survey developers need to make a tradeoff between consistency and redundancy to obtain validity [73]. With these considerations in mind, we included a number of retest questions for validity, cf. Section 3.1.8. and Table 2. The initial inclusion of five retest questions made the survey too long. Inconsistency for pretest 1 was rather high, with an individual CR of 0.45. Therefore, based on the considerations proposed by Ozdemir [73], we decided to reduce the number of retest questions and, consequently, the length of the survey. Only two participants (pretests 2 and 4) during the subsequent pretest interviews answered completely opposite to their previous choice at the level of criteria. The remaining participants chose the correct side on the AHP rating scale, with some deviations in assigned values. However, a solid assessment of this issue would require a larger sample size, such as that in a study by Brod, Stewart, Sands and Walton [74], who developed a simple dementia QoL instrument (DQoL), which was tested on 99 participants. Nearly all participants were able to respond to questions appropriately, suggesting that people with mild-to-moderate dementia could be considered good informants of their own subjective states, paving the way for consideration of patient responses as the gold standard for assessment of QoL in PlwD [74]. Individual CRs at the level of criteria ranged from 0.08 to 0.86, with a consolidated CR of *n* = 11 PlwD of 0.37 (0.038) based on the arithmetic (geometric) mean for aggregation of individual priorities (judgements). With a strict consistency threshold of 0.1, as suggested by Saaty [63], we would have had to exclude *n* = 9 participants from the analyses. Application of the generally accepted cutoff at 0.2 [57,75,76,77] would still have resulted in an exclusion of *n* = 7 participants from our analyses. As noted by Goepel [60], the application of the ten-percent rule and even the twenty-percent rule may be too strict for certain practical applications. Particular circumstances may warrant the acceptance of a higher value—even as much as 0.3 [64]. Furthermore, the achievement of low inconsistency should not be the only goal of the decision-making process. Reasonable consistency is necessary but not sufficient for a good decision [78]. A cutoff at inconsistencies above 0.3 would have resulted in *n* = 5 participants excluded from our analyses, i.e., slightly less than half of the total sample. One option also was discussed by Danner et al. [55] is to ask participants with high inconsistencies to reconcile their judgements. However, and similarly to Danner et al. [55], who also surveyed an aged and sometimes cognitively impaired patient group, we observed that participants became confused when their choices were questioned by the interviewer, as they thought they did not “perform” as they should have. A practical obstacle is the use of a paper-and-pencil survey, which does not allow for immediate and accurate calculation of CRs and subsequent query of the participants to revise their judgements. Additionally, high inconsistencies may have been caused by an inappropriate use of ‘extreme judgements’, as also noted by Danner et al. [55]. However, this assessment requires a larger sample size than that used in the present study and should be examined in future research, as planned for the main study in the overarching *PreDemCare* study [37,38]. No standard for sample size determination exists for AHP surveys. Hence, sample size for the planned main study was likewise to IJzerman et al. [75] oriented in sample size determination for conjoint analysis. A detailed description of this method lies outside the scope of this report but can be found elsewhere [37].

One of the most considerable challenges was to find an appropriate display of the AHP rating scale that would be well understood by PlwD. The research team considered the layout suggested by Danner et al. [55]. However, we perceived the layout as too abstract for PlwD to relate to. Apart from the visual layout, the number of answer options was discussed among the research team based on feedback from EP1 and patients, as well as recommendations for the design of dementia-friendly surveys to preferably not include more than three answer options for each question [30]. Because the layout displayed in Figure 3 was well-received by the pretest participants, we decided to keep this version of the rating scale, i.e., a simpler visual layout than that proposed by Danner et al. [55], but to keep the main answer options (1, 3, 5, 7 and 9) of the original AHP rating scale [56].

When PlwD struggled with which perspective to apply during completion of the AHP survey, the assisting interviewer, W.M., instructed the to apply “today’s perspective” and emphasized that preferences may change in the future. Some authors argue that patient preferences need to remain stable over time to be reliable [79]. However, as noted by van Haitsma et al. [80], preferences are based on the processing of needs, values and goals and therefore may shift as the social environment or contextual circumstances change. Particularly during the ongoing COVID-19 pandemic, many contextual circumstances for this patient group, such as access to social activities, may change. Hence, we conducted pretest-interviews under recognition of van Haitsma et al. [80].

### Limitations

Our study has several limitations. The overall sample sizes (*n* = 11 PlwD, *n* = 4 DCMs, *n* = 4 physicians) of this current *formative* qualitative study were small compared to usual sample sizes in *general* qualitative research. However, as previously mentioned (cf. Section 2.5), we followed the guidelines presented by Hollin et al. [28], who emphasized that sampling should not focus on the number of units, but actionable input for the development process, i.e., a diversity of perspectives. Hence, sampling adequacy in formative qualitative research, such as the current study, may include smaller samples than general qualitative work, which, based on the limited study purpose, can be viewed as adequate [28]. Aside from Hollin et al. [28], we oriented ourselves in the existing literature on quantitative patient preference research for expected saturation point, including previous research by the second author, A.R., who reported similar sample sizes in the *formative* pre-study phase(s) [45,46,47,48,49,50]. The complete *formative* pre-study phase in the overarching *PreDemCare*-study [37,38] included two subphases: qualitative interviews for (sub)criteria identification including *n* = 10 PlwD and *n* = 2 DCMs, i.e., a total of *n* = 12 participants (Stage 1) [31], and the current study for pretest and design of the AHP survey(s) including *n* = 4 DCMs, *n* = 11 PlwD and *n* = 4 physicians, i.e., a total of *n* = 19 participants. The complete formative prestudy phase therefore included *n* = 31 participants, which is similar to sample sizes reported in general qualitative research [33]. By including different stakeholders, we ensured a diversity of perspectives for provision of actionable input, as emphasized by Hollin et al. [28]. A question remains at to whether the choice of qualitative research methods in the development of a quantitative preference instrument suffice to provide actionable input. However, Hollin et al. underlined that the use of formative qualitative research in the developmental phase is essential to ensure both face and content validity [28]. Furthermore, as recommended by the Alzheimer’s Society UK [30], it is important to pilot and consult with PlwD themselves in survey instrument development. Based on our experience on site with this patient group, we deemed it necessary to conduct interviews with the “thinking-aloud technique” [32] to capture PlwDs’ thoughts as meticulously as possible and ensure comprehensibility of the final quantitative preference instrument. By including patients, as well as clinical experts, in different stages of the overall pre-study phase, we aimed to increase the face and content validity of the final instrument [28].

An often-mentioned limitation of patient preference studies is heterogeneity in the surveyed populations. Depending on certain characteristics, patients may respond differently to specific interventions and differ in terms of how they value particular attributes of interventions [81]. This may be particularly true for an aged and often multimorbid patient group, such as PlwD [82]. To account for potential heterogeneities in preferences in both populations, we included extensive self-developed sociodemographic questionnaires for subsequent subgroup analyses. In contrast to other methods, which only allow for analysis of aggregated data, the AHP allows for evaluation of preferences on an individual basis [24]. This information can then be used to assess heterogeneity. The advantage of the AHP method is that both group decisions and individual decisions are possible.

As in previous patient preference studies [55], we conducted a pretest survey with interviewer assistance. The survey had to be interviewer-assisted, as most participants had visual impairments and needed assistance with reading. Danner et al. [55] conducted 10 surveys with two interviewers. Attendance of one or two interviewers was discussed extensively among the research team and during EP1. Previous qualitative interviews [31] showed that attendance of two interviewers made the PlwD more nervous, which was also confirmed by the DCMs during EP1. Hence, we decided to have only one interviewer (W.M.) assist during the pretest surveys. W.M. strictly adhered to the standardized interviewing procedure presented by Danner et al. [55] and, prior to pretests, was trained with members of the researcher team and study nurses on site with extensive experience in interviews with PlwD. These considerations should be reflected upon prior to future patient preference research with an aged and cognitively impaired patient group.

With regard to the requirements of credibility and dependability as criteria of trustworthiness, we remained compliant with our research focus and collected a manageable amount of data in a short period of time, which enhanced our study’s trustworthiness [83]. However, the transferability of findings is limited due to the rather small sample sizes of included subjects, the specificities of our setting and respective cultural differences. Still, due to the rigor in the methodological process and report, we consider our findings trustworthy.

## 5. Conclusions

Our study provides initial data from a pretest study of a dementia-friendly AHP survey. Extensive technically descriptive sentences of the (sub)criteria should be avoided. Validity and consistency of responses may pose a challenge that requires consideration about an appropriate CR threshold. Layout and presentation of the AHP scale not immediately intuitive for an aged and cognitively impaired patient population may be a challenge but can be addressed by creativity among survey developers. The heterogeneity of an often multimorbid, aged and cognitively impaired patient group should be considered by inclusion of sociodemographic questionnaires for potential subsequent subgroup analyses. Assistance during completion of an AHP survey with an aged and cognitively impaired patient group is required. Consideration of our findings may contribute to content and face validity, as well as internal consistency, which still needs to be tested with a larger sample size. Our detailed process report may increase reproducibility in future preference research on dementia with application of quantitative preference instruments.

## Figures and Tables

**Table 1:** Patient characteristics (*n* = 11).

Characteristic		*n* (%)
Age	60–71	2 (18.2)
	71–80	4 (36.4)
	81–90	3 (27.2)
	>90	2 (18.2)
Gender	Female	6 (54.5)
	Male	5 (45.5)
Family status	Married	6 (54.5)
	Widowed	4 (36.4)
	Divorced or separated	1 (9.1)
Highest educational degree	No degree	1 (9.1)
	8th/9th grade	1 (9.1)
	10th grade	2 (18.2)
	Degree from a technical/vocational college	4 (36.4)
	Degree from a university of applied sciences or university	3 (27.2)
Previous occupation [65]	Skilled worker	2 (18.2)
	Employee with limited decision-making powers (e.g., cashier)	6 (54.5)
	Lower grade with high qualification in employment (e.g., doctor, professor, engineer)	3 (27.2)
Monthly net income	EUR 1001–1500	1 (9.1)
	EUR 1501–2000	3 (27.2)
	Not known	3 (27.3)
	Prefer not to say	4 (36.4)
Stage of cognitive impairment _a_	Early	9 (81.8)
	Moderate	2 (18.2)
Non-pharmacological treatment		7 (63.6)
	Memory work (e.g., memory exercises, rehabilitation)	2 (28.6) _b_
	Occupational therapy	2 (28.6) _b_
	Physical training (e.g., physiotherapy, sports groups)	7 (100.0) _b_
	Artistic therapy (e.g., music therapy, art therapy, dance therapy, theater therapy)	1 (14.29) _b_
	Other (speech therapy)	1(14.29) _b_
Self-rated general health	Good	5 (45.5)
	Satisfactory	5 (45.5)
	Less good	1 (9.1)

_a_ Determined by study nurses as gatekeepers based on latest assessment with a validated tool (MMSE) [52] in the clinical trial from which the patients were recruited. _b_ Percentage calculated based on those (*n* = 7) who indicated that they received non-pharmacological treatment. Multiple selections possible.

**Table 2:** Results of retest questions among PlwD.

	PT1	PT2	PT3	PT4	PT5	PT6	PT7	PT8	PT9	PT10	PT11
Examples											
No social exchange	1/9	1/3	1/3	1/7	1/5	1/6	1/5	1/5	1/3	1/3	1/5
A lot of social exchange	9	3	3	7	5	6	5	5	3	3	5
Social relationships	9	N/A	N/A	N/A	N/A	N/A	N/A	N/A	N/A	N/A	N/A
Additional cost	1/9	N/A	N/A	N/A	N/A	N/A	N/A	N/A	N/A	N/A	N/A
Criteria											
Social exchange	9	1/5	**3**	**5**	**5**	5	5	9	3	1	**6**
Physical activities	1/9	**5**	1/3	1/5	1/5	1/5	1/5	1/9	1/3	1	1/6
Social exchange (2)	7	**3**	**7**	1/6	**1**	5	5	9	3	1	**7**
Physical activities (2)	1/7	1/3	1/7	**6**	1	1/5	1/5	1/9	1/3	1	1/7
Social exchange (3)	9	N/A	N/A	N/A	N/A	N/A	N/A	N/A	N/A	N/A	N/A
Physical activities (3)	1/9	N/A	N/A	N/A	N/A	N/A	N/A	N/A	N/A	N/A	N/A
Sub-criteria											
Indirect contact	1/9	1/3	1/5	1/6	1/5	1/3	1/5	**6**	1/3	1	7
Direct contact	9	3	5	6	5	3	5	1/6	3	1	1/7
Indirect contact (2)	1/9	1/3	1/5	1/6	1/5	1/3	1/5	**7**	1/3	1	7
Direct contact (2)	9	3	5	6	5	3	5	1/7	3	1	1/7

Notes: The retest questions “*Social relationships* vs. *additional cost*” and “*Social exchange* vs. *physical activities* (3)” were removed after the first pretest interview to reduce the length of the survey. The bold numbers indicate a difference in first vs. retest judgement. Abbreviations: PT = pretest.

**Table 3:** (**a**) Overview of individual consistency ratios for PlwD at the level of criteria. (**b**) Consolidated consistency ratio for group decision among PlwD (criteria level).

(a)			
PT	Principal Eigenvalue	GCI	CR (in %)
**1**	8.826	1.46	45.1%
**2**	7.265	0.7	20.2%
**3**	7.795	0.96	28.6%
**4**	9.271	1.67	52.2%
**5**	6.599	0.35	9.6%
**6**	11.382	2.63	85.9%
**7**	9.574	1.77	57.0%
**8**	7.631	0.88	26.0%
**9**	6.819	0.46	13.1%
**10**	6.504	0.29	8.0%
**11**	9.814	1.85	60.8%
**(b)**			
**Consistency *n* = 11**			
Principal Eigenvalue			6.237
CI			0.14
CR (in %), GM			3.8%
CR (in %), AM			36.9%

Abbreviations: CR = consistency ratio, GCI = geometric consistency index, PT = pretest, CI = consistency index, GM = geometric mean, AM = arithmetic mean.

## Data Availability

Data and methods used are presented in sufficient detail in the paper so that other researchers can replicate the work. Raw data will not be made publicly available to protect participant confidentiality.

## Supplementary Materials

The following supporting information can be downloaded at: https://www.mdpi.com/article/10.3390/ijerph19148554/s1, File S1: Main categories with sub-categories and respective key quotations from expert panels and individual pretest interviews; File S2: Partial Transcript Pretest—Interview 8.
