# Supplementary material for: Development of a Quantitative Preference Instrument for Person-Centered Dementia Care—Stage 2: Insights from a Formative Qualitative Study to Design and Pretest a Dementia-Friendly Analytic Hierarchy Process Survey
Source: Int J Environ Res Public Health. 2022 Jul 13;19(14):8554. doi: 10.3390/ijerph19148554 (PMC9321359; doi:10.3390/ijerph19148554)
Supplement: Supplementary file 1 [file ijerph-19-08554-s001.zip › Supplementary Materials File S1.pdf]

Supplemental Table S1. Main categories with sub-categories and respective key quotations from expert panels (EP1: N=4 DCMs, EP2: N=4 physicians) and individual pretest-interviews (PT) (N=11 PlwD & N=3 informal CGs)

CONTENT	**1) Title page with introduction (PlwD):**<br>Emphasize that the survey asks about the PlwDs opinion and is not a test.	(1) I wrote this [in bold and with exclamation point], because during the [previous] interviews they were a little stressed. […] they thought that I was going to test them and I always had to say "I'm not testing you, I'm just asking you what you wish for." *(I1, EP1, 00:12:45)*<br>(2) [Confirmed by members of EP1]
	**2) Title page with introduction (Physicians):**<br>Dementia status.<br>Proxy perspective vs. physicians' judgements.	(1) However, it is quite interesting that there can also be changes in preferences during the progression of dementia diseases. So even [people who like to be by themselves] suddenly become [fond of being members of groups]. So this is possible, in the advanced stage, however. I don't know if [the colleagues] have also experienced this, but these are things that I have experienced with my patients. *(D3, EP2, 00:10:52)*<br>(2) I did not really understand which stage of dementia I should imagine during completion of the survey. *(D2, EP2, 00:12:46)*<br>(3) Well, I would say...I have really tried to put myself in the patient's shoes. But of course you have to realize that every patient is different and if you know the patient, then you sometimes decide this way and sometimes that way. So, from my point of view, it is less accurate to choose this parameter [to complete survey from proxy perspective] than to say I imagine it "for" my patient [expert opinion, i.e. physician judgement]. I think that [physician judgement] is easier in the end. But I did it exactly like it was instructed [on the title page of the survey], but at some times I was sitting in front of it [the survey] and thinking hm hm hm...so it was not completely consistent at all times. *(D4, EP2, 00:25:15)*<br>(4) Well, I think these [proxy perspective vs. physician's judgement] are two different things. One is what you would call the Theory of Mind, so we put ourselves in someone else's shoes and try to answer [the survey] from a general patient perspective. And the other would be the concrete case of an individual patient, where we then act as a proxy. [...] So I think we should be clear about this. Do we want to know about the doctor's point of view or do we want the other point of view...that we should act as patient advocates. I think that is a bit diffuse. *(D3)* [...]<br>(5) So I think it would be more interesting, but of course that is a question of methodology, what is required [preferred] from the physician's point of view? Then you would have two groups. And then the question would also be quite clear. So what do we as physicians think is important [for PCC of community-dwelling PlwD]. That could be clearly formulated. And the other [question] would be what is important from the patient's point of view. One is a professional question and the other is a question of the Theory of Mind, so we put ourselves in the other person's shoes. *(D3, EP2, 00:28:50)* [...]<br>(6) So I think if you ask that you would like to know from the physicians what they think "about" their patients [expert opinion], those are harder facts than if you expect someone to put themselves in someone else's shoes. *(D3, EP2, 00:31:22)*<br>(7) […] I would also find it clearer as [D3] just said and additionally, the comparison [patient preferences vs. physician's judgement] is actually interesting. Whether the PlwD have similar tendencies [preferences] [as we experts]. And these differences are interesting above all. If some of the doctors find things important, which are not so important for the patients, it is good to know that. *(D2)*
	**3) Description of (sub)criteria:**<br>Abstract titles need to be described by concrete examples the PlwD can relate to, list examples throughout the complete survey.	(1) "Social relationships"...eh I can't figure out now what this involves. [Laughs] *(P, PT2, 00:29:26)*<br>(2) If you want, you can... *(I1)*<br>(3) Memory exercises, but I know that... *(P)*<br>(4) Have a look...*(I1)*<br>(5) "Social relations"...I don't know about that...*(P)*

	(6) [Turns pages back to list of criteria with examples] You have the list here. *(I1)*<br>(7) Oh, it's up there...[laughs]. *(P)*<br>(8) You can look at it again. *(I1)*
**4) Formerly merged criteria demerged:**<br>'Attention & support with worries, feelings and memories' demerged from 'Social relationships'.	(1) [Reads] "Attention and support with worries and feelings." Oh. I don't know what that is...attention? *(P, PT2, 00:06:32)*
**5) AHP Axiom 2:**<br>Homogeneity of criteria.	(1) I had a bit of difficulty...with comparing two variables, […] for example, "Memory Exercises" vs "Organization of the health care system"...for me, these are two things that cannot be compared directly. So I had a little bit of difficulty there or you can't really prefer one of the two things, from my point of view. […] I had a little bit of difficulty to evaluate that. *(D1, EP2, 00:01:58)*<br>(2) […] This criterion, "Organization of health care", we have actually discussed [a lot] […] we also talked with the PlwD, and as much as the title [of the criterion] may seem very abstract, it is then through [the explanation/ definition], so "the location of doctors, pharmacies, etc." and "the conversation [among the health care providers and with the patient]" [clarified]. The content of the criterion pulls it down again to such a concrete level for the PlwD that they [show preferences] to it. […] you always have to see it in such a way that you see the criteria in a cluster-relationship […] about "what is important to me for my care" [as a community-dwelling PlwD]. And in this regard it is also important where do I have to go to the doctor, is he right next door, is everything under one roof, how am I included in the conversation. And when I checked again with the [PlwD] themselves [and their pretest results], in the [pairwise comparison] of "Memory exercises" vs. "Organization of health care", four said "equally important," and the rest actually expressed a preference for "Organization of health care". *(I1)*<br>(3) Four out of how many? *(D2)*<br>(4) In the pretests, we had 11 people. *(I1)*<br>(5) Because "equal[ly important]" – I chose this every now and then out of desperation, when I didn't actually think it was comparable. So how can you compare "solving crossword puzzles" with "my doctor is just around the corner". [I] find that difficult. *(D2)*<br>(6) So I discussed this [earlier with a colleague], and I said that it actually is a matter of preference […]. There are people who say "I want to deal mainly with my exercises and where I get help that's not really important to me." Or the other way around [others] say "Oh, these stupid memory exercises, they annoy me, I want to have good accessibility [to health care]," for example. I can also understand what was just said [by D1 & D2]. I also had a bit of a hard time with it, but then I always tried to see the point of view of the PlwD […]. *(D3)*
**6) Introduction of sub-criteria:**<br>Initially description for criteria, i.e. examples, kept above introduction of sub-criteria, which caused confusion and subsequently was removed.	(1) [Reads explanation for criterion "Social relationships" and related sub criteria] Social relations...conversations, writing letters, telephoning, meeting neighbors...[reads all examples again]...? *(P, PT2, 01:00:32)*

	**7) Appropriateness of (sub)criteria:**<br>Refinement of sub criteria for *1) Social Exchange, 2) Physical Activity, 3) Memory Exercises,* and *6) Organization of Health Care* after EP2.	(1) I wanted to make a comment about this [criterion] *"Organization of health care"*, […]. I thought that was very tendentious. I actually don't understand this juxtaposition. […], I experience it differently than it is written here. […] in such a way that the patients, whom I see over many years, appreciate it to have their general practitioner, even if they must [travel longer] there. […] And I do not understand, why "shared decision-making" […] should not be possible in decentralized structures. I experience the opposite to be true; if I have known the patient for a long time as an individual [practice based] physician and […] there will always be a shared decision, if it is somehow possible. […] I think this [presentation] [...] whether rather paternalistic decisions are made or shared decision-making takes place, is not dependent on whether there is a decentralized or central structure of care. *(D2, EP2, 01:04:43)* […]<br>(2) […] Regarding care organization [the PCC-literature] discusses very much about "integrated care". [And our idea to describe "integrated care" in a laypeople way was]; I have centralized structures and because [the services] are closer together, I also [gain increased] shared decision-making. We also discussed this with the PlwD, […] I think it [is] also [influenced] a bit [by] the setting, [where] we are here […] in the former GDR, […]. And [regarding the keyword] "polyclinics" [the response was] for example; "Yes, that was good, everything was under one roof and people [the health care providers] also talked more to each other". I think that is why [criterion 6 "Organization of health care"] developed the way it looks now. [During the pretests] I always asked again "Okay, why did you now choose [that sub criterion] in this comparison – was it because of the structures, so where you find your doctor or because of the communication [shared decision-making]?" And then the reply [is] "Both is important!" *(I1, EP2, 01:07:25)* [...]<br>(3) But of course, you have to be careful... by asking this question...you imply something […] – that decentralized structures [just] send out […] reports on findings [and do not communicate with each other]. That would be the decentralized specialist physician system. If that still [is like this in reality] to be honest, I don't know at all. [But] what is implied by this [sub criteria for "Organization of health care"] is that the physicians do not talk to each other when they are [located] in individual practices. Whether that is really the case, I cannot say. For my part, I can only say that we *do* talk to each other, even if we are in individual practices. *(D3, EP2, 01:09:36)*<br>(4) Yes, talking to each other or not talking to each other is possible in both structures. *(D2)*<br>(5) Yes, that is what I mean and that is why this linkage between "doctor decides without involving the patient" and "decentralized structures" is problematic. Not so much the structure itself, but the linkage. It may well be that the [PlwD] in the (new) eastern states of Germany [former GDR] see it that way, but we [do now have] the MVZs [community health centers] here...and I cannot say that I didn't coordinate with the GPs in the past. *(D3)*<br><br>(6) Did you find that [the changes in sub criteria] understandable or was that somehow [difficult]? *(I1, PT12, 00:21:38)*<br>(7) Nah that was [al]right yes. *(P)*<br>(8) […previously] we had asked with [regard to the sub criteria for] "Social relationships"; "What is more important to you, indirect or direct contact?". But the experts complained that [portrayed in this way it portrays] better and worse [an ordinal scale]. We then […] spoke again [in the team and thought] that it would also be interesting to know whether aged people always prefer only family and friends or whether some perhaps also like to meet new people? *(I1)*<br>(9) Yes, but that is a question of how the person [and the personality] is! *(P)*

		(10) So we have changed that [criterion 2 - physical activities] now [after the expert panel with the doctors]. Before I had asked "Alone or in a group", now I would ask [...] the format, so alone or in a group [...] or the place. Do you prefer to do it at home for yourself, do gymnastics for example, or do you prefer to go out? *(I1, PT13, 00:09:32)*<br>(11) Yes, I bought myself a pedal trainer, because I can't go outside anymore. *(P)*<br>(12) Exactly or do you like to go out into nature...there are also people who like that. *(I1)*<br>(13) Yes, I would like to...with my acquaintances or my children here or so. But I can't walk that far anymore. *(P)*<br><br>(14) With the memory exercises [criterion 3], you can do leisure activities. Here, for example, we have someone who reads or you love to do crossword puzzles […] Or some like to learn something new, for example an instrument or a language or something new to cook. Just something new. You said earlier that you would like to see something new, maybe you would also like to do something new? *(I1, PT13, 00:10:56)*<br>(15) I would like to go shopping at times. I would like to see the mall...so my big [son] calls from the mall and says "Yeah what else do you need?" and then I have to say from up here what I need when I go to the mall. [...] *(P)*<br><br>(16) And then finally in the "Organization of health care" [criterion 6]. We have now stated on the one hand [sub-criterion 1] "communication" [...] between doctors and therapists, but also […] that you are included is important...*(I1, PT13, 00:13:10)*<br>(17) Hmmm [nods in agreement]. *(P)*<br>(18) ... or but of course also important is the location of things [sub-criterion 2]. *(I1)*<br>(19) Yes! [...] Not everything has to be big, but if I go to the corner [of the city], then I know that there is an orthopedist, a physician [general practitioner], maybe an ophthalmologist, a pharmacy and so on. And there's the other one back there again, then I go to that side. But I have my pharmacy with me and...*(P)*<br>(20) But the polyclinics at that time…*(I1)*<br>(21) Yes! Yes, yes, we had that and it is still partly like that. […] Yes, but it's good that they didn't take them away. *(P)*<br><br>(22) Oh that's hard [sub criteria for criterion 6]. Both are important! That's yeah...both are important. *(P, PT13, 00:25:08)*
	**8) Validity and inconsistency in the AHP-survey:**<br>Inclusion of re-test questions.	(1) Yeah but for them it's like a test...they're then surprised when you ask the same thing multiple times. [...] But try it out. [...] *(DCM2, EP1, 00:53:35)*<br>(2) [Confirmation by EP1]<br>(3) Let's see. Maybe I'll take some of these [re]test questions out again. *(I1, EP1, 00:56:25)*
	**9) Heterogeneity of respondents (PlwD):**<br>Remarks from Expert Panel 2 to keep this particularly in mind for this patient group.	(1) That applies to many things. So for example this [sub criteria for criterion 2]...here one does sports alone on the pictogram and the others find it nicer in the group. There are all kinds of things that influence this... how was the person before and how much of his personality is still preserved? If you were a loner before, why should you suddenly want to do gymnastics in a group when you have dementia? *(D2, EP2, 00:08:58)*

	**10) Sociodemographic questions (PlwD):**<br>Living situation of patients may influence their preferences.	(1) […] These are obviously the things that were important to the [PlwD]. But I actually missed a category...there was always talk of domesticity, but "how to live" was missing for me completely. Do I want to continue to live in the home, do I want to live in a shared apartment, do I want to live in assisted living, etc.? I was a bit surprised that this didn't appear anywhere. Did that get discarded beforehand, or? *(D2, EP2, 00:14:00)*<br>(2) We actually assumed that everyone knows what is meant by domesticity. [...] So we mean people who are not yet institutionalized, that is, not yet living in a nursing home with permanent care. *(I1)*<br>(3) Hm. But there will certainly be differences. […] *(D2)*
FORMAT	**11) Outline / structure of the complete survey:**<br>Restructuring of usual setup of an AHP-survey to accommodate special needs of patient group.	(1) And we have already moved the sociodemographic questions to the end [of the survey], because the problem with this survey is that it is very long, and the problem is of course that the [PlwD] get tired. And the sociodemographic questions is something that is quite easy to [answer]...*(I1, EP1, 00:13:45)*<br>(2) [Agreement from EP1.]
	**12) Sequence of criteria-related pairwise comparison questions:**<br>Row by row vs. diagonal line wise.	(1) But we've already had that [criterion]. *(P, PT1, 00:58:53)*<br>(2) Nah, we haven't had that comparison yet. *(I1)*<br>(3) Oh, really? *(P)*
	**13) Length of survey:**<br>Keep the length of the survey limited and comprehensible.	(1) All right. One hour and 22 [minutes]...I have to shorten the questionnaire. *(I1, PT2, 01:22:58)*
	**14) Formatting:**<br>E.g. use of page-numbers, font, font size, self-reading vs. reading up for patient, decrease visual stimuli.	(1) And with the pages...now you've already said "Whew, 37 pages" - so maybe you'd find it better if that wasn't there, so it wouldn't seem so scary? *(I1, PT1, 00:01:15)*<br>(2) [Laughs] Maybe so. [Laughs.] *(P)*
	**15) Layout:**<br>Transformation of AHP rating scale.	(1) Do you find, for example, do you find that this is too exhausting with all the numbers? Would it be better if it was just 1, 5, and 9, for example? So if we said that's just like this [covers 3 and 7]? *(I1, PT1, 00:19:19)*<br>(2) Yeah...*(P)*<br>(3) Would that be easier? *(I1)*<br>(4) Maybe... [hesitant]<br><br>(5) [Draws the ranking scale as an actual scale and weights, cf. Fig. 3] So this is a scale...it's got this... here. *(I1, PT2, 00:26:43)*<br>(6) Right. (P)<br>(7) And then we have 9...so 1, 2, 3, 4, 5, 6, 7, 8, 9 weights here. And on these two trays there are always two different things to choose from. And you can then sort of always balance with the weights how much it [a characteristic] is more important to you. *(I1)*<br>(8) Hm exactly. Yes yes. *(P)* […]<br>(9) Might that be a little more intuitive? *(I1)*<br>Hm. Yes that's good! *(P)*

	**16) Explanation for survey procedure:**<br>Procedure of choosing one (sub)criterion in pairwise comparisons oriented in example from Danner et al. [55].	(1) [Reads]... "Example: Below the criterion 'Social relationships' is compared with the criterion 'Support with everyday activities'. Please indicate whether the left or the right criterion is more important, and by how much."...what's uhm supposed to...? *(P, PT1, 00:11:25)*<br><br>(2) [Looks at example with choice of a side dish] Oh! [Laughs in amazement.] *(P, PT2, 00:22:40)*<br>(3) Now comes a very general example. *(I1)*<br>(4) [Reads loud] You are in a restaurant and you have to choose between two side dishes, rice or potatoes...[Reads example.] *(P)*<br>(5) Do you understand this example? *(I1)*<br>(6) Yes! *(P)*
	**17) Simplification of pairwise comparisons:**<br>Use of *"A or B"* or *"Left or Right"* to ease the task of pairwise comparisons.	(1) [I have a] proposal. If I now had A standing here [left criterion] and B here [right criterion] and I would now ask "A or B"? Would that be easier for you? For the decision? Or would that make no difference? *(I1, PT1, 00:57:35)* […]<br>(2) There I would say...A is already important! *(P)*
	**18) Assistance during interviews:**<br>Two vs. one interviewer to assist.	(1) Well then. I'm assisting. And maybe [a colleague] will come along too […]. *(I1, EP1, 00:22:45)*<br>(2) I think it's better if you go alone - don't take so many people with you! Two people make them more nervous again. *(DCM2)*
	**19) Perspective in pairwise comparisons:**<br>Assist PlwD with general problem of which perspective should be applied during completion of survey (today vs. in the future).	(1) [Laughs] But [...], if you had to decide now...you can also say it's equally important, but maybe one is a bit more important than the other? What do you think? […] *(I1, PT1, 00:36:50)*<br>(2) Well, it always depends on the situation, doesn't it? *(P)*<br>(3) But that can change...you can also say "today I have the feeling that this is more important to me"...but that can also change. You can always ask about it. But today...if you think today like this... "Ok, today I think this is somehow more important to me" or..." *(I1)*<br>(4) Yes it is. Because making phone calls...*(P)*<br>(5) That's more important to you today? *(I1)*<br>(6) Yes. *(P)*<br><br>(7) For the social relationships there I have to do a little bit...and if [the situations worsens], then maybe I have to have a little bit of support with everyday activities. *(P, PT2, 00:33:08)*<br>(8) But right now today. If someone would ask you now "what would be very important for you today for your care? That you get support with everyday activities or that you can talk to someone, stay in contact?" […] *(I1)*<br>(9) I mean, you always need social relationships to keep you going. And if you no longer [are physically fit enough] […] you would also need support. […] I just read "a little more important". I would give [3] to social relations. *(P)*

LANGUAGE	**20) Find words for (sub)criterion understandable for PlwD:**<br>Consider usual German translation and find respective easier understandable synonyms.	(1) I always refer to the [criteria] as "characteristics" [German: Merkmale]. But I was thinking, maybe there's another word that describes it better. So I had now specifically thought about "components" of care or areas. What do you think? Or do you think "characteristic" is a good word? You can say that, too. *(I1, PT4, 00:05:00)*<br>(2) For us old people it is. For us, that's...*(P)*<br>(3) Characteristic is all right? Can I leave it like that? *(I1)*<br>(4) Hm [nods]. For us old [people], it's understandable. *(P)*
	**21) Avoid long sentences:**<br>Concerns both informative sentences on the title page, introduction, but also questions on top of each pairwise comparison.	(1) So and what do we have now? [Reads] Which care characteristic is more important to you and to...? *(P, PT1, 00:38:43)*<br>(2) It is always the same task [question]. It's the same task every time on every page now. *(I1)*<br><br>(3) This is really difficult here. [Reads sentence aloud.] *(P, PT2, 00:00:01)*<br>(4) Do you think it's too long? *(I1)*<br>(5) Yeah... such long sentences...that is, that is...*(P)*<br>(6) Yeah would you like to have it shorter. [Reads out sentence] That was the one, wasn't it? *(I1)*<br>(7) Yeah, yeah. *(P)*<br>(8) Yeah okay. That's a very long one. Good feedback. I'll shorten it. *(I1)*

	**22) Choice of words matters:**<br>Consider needs of this patient group, pay attention to their reactions – also about seemingly unimportant words.<br>Avoid “foreign” words like “activity”, “transparent” etc.<br>Social contact / social relationships / social exchange.	(1) [Reads]...the place...placement of physicians...[reads]. *(P, PT1, 00:09:31)*<br>(2) The "placement"...you stalled a bit there, didn't you? At that word. *(I1)*<br>(3) Yes yes...[...] *(P)*<br>(4) ...would that possibly be a better word if it said "location" of physicians...? *(I1)*<br>(5) Yes, yes! *(P)*<br><br>(6) On social relations...eh there I don't get it now, what falls under this. [Laughs.] *(P, PT2, 00:29:26)*<br>(7) If you want, you can...*(I1)*<br>(8) Memory exercises, but I know that...*(P)*<br>(9) Take a look...*(I1)*<br>(10) "Social relations"...I can't think of it now....*(P)*<br><br>(11) [Reads out] First characteristic: social contact. Examples would be: Talking, writing letters, talking on the phone, meeting friends or neighbors, meeting in the club room of an assisted living facility, pets, family. *(I1, PT3, 00:02:49)*<br>(12) All right! *(P)*<br><br>(13) Would you rather call it "social relationships" or "social exchange"? Which do you think sounds better? *(I1, PT12, 00:27:54)*<br>(14) Relationships...exchange...[mumbles]...exchange I would say then! *(P)*<br><br>(15) Would you find it better...I was thinking about that earlier when I printed out the questionnaire. I was already thinking about whether it would be easier to say "organization of health care system"...because somehow more people can relate to that than if you just say "care". Because it's about the location of the doctors for example...Because that's more the health care system.... *(I1, PT4, 00:18:20)*<br>(16) Hm hm hm. [nods in agreement]. *(P)*<br>(17) Do you think that would be easier if you say "health care system" rather than "care"? *(I1)*<br>(18) I think so. [nods]...More understandable for our generation. *(P)*

	**23) Use of ICONs as visual aids:** Consider appropriateness / comprehensibility of ICONs to ensure that this visual language helps.	(1) How do you find the pictures? […] This is supposed to represent that you sort of...that not everything is in one place like here in the polyclinic, but that you have to go to many different places. Is that understandable or is that too complicated? *(I1, PT1, 00:30:20)*<br>(2) Yes that is...so here I would have honestly [said] I wouldn't have [known what is meant by that]. *(P)*<br>(3) Okay. And how would that be if I placed several little houses here like this and then always made arrows like this [from the center] there? Would that be easier to understand? *(I1)*<br>(4) Yes. Here [regarding first draft of ICON for sub-criterion 1 to Criterion 6] I wouldn't have been able to do anything now. […] *(P)*<br>(5) Hm and do you find that this [first draft ICON for sub-criterion 2 to Criterion 6] is well mapped with the hands that you have so to speak together...do you find that good or not so good? *(P)*<br>(6) Joaa that's already good...but I wouldn't have understood so much with the three hands here now [laughs]. […] *(P)*<br>(7) Would it perhaps be nicer to have a doctor and a patient pictured here, who are in conversation? *(I1)*<br>(8) Yes, yes! I wouldn't have been able to do anything with the three hands now. *(P)*<br><br>(9) Hm. But you can understand this with the different shapes? That's understandable? *(I1, PT2, 01:05:04)*<br>(10) Yes. *(P)*<br>(11) And the pictures [new ICONs] you also find understandable? *(I1)*<br>(12) Yes. *(P)*

Abbreviations: CG = Caregivers, D1-4 = Doctor 1-4, DCM = Dementia Care Manager, EP1 = Expert Panel 1, EP2 = Expert Panel 2, I1 = Interviewer 1, P = Patient, PlwD = People living with Dementia, PCC = Person-Centered Care, PT = Pretest
