# Supplementary material for: Development of a Quantitative Preference Instrument for Person-Centered Dementia Care—Stage 2: Insights from a Formative Qualitative Study to Design and Pretest a Dementia-Friendly Analytic Hierarchy Process Survey
Source: Int J Environ Res Public Health. 2022 Jul 13;19(14):8554. doi: 10.3390/ijerph19148554 (PMC9321359; doi:10.3390/ijerph19148554)
Supplement: Supplementary file 1 [file ijerph-19-08554-s001.zip › Supplementary Materials File S2.pdf]

Supplemental File S2 – Partial Transcript Interview 8

Example for questions related to criterion 6 "Organization of the health care system" and related sub-criteria.

From 00:43:20

Interviewer (I): The organization of the health care system. We have just [read], you can have these decentralized structures, so that you have all these individual practices and the doctor decides without involving the patient or you have the central structures such as in the polyclinics and the medical centers, so everything [is] under one roof, and the doctor decides together with the patient. Which would be more important to you?

Patient (P): I do see my General Practitioner (GP), but I still think it's good in general.

I: So that it is all under one roof is good, so also the pharmacy, etc.

P: Hm yes, so like the clinic. Then you can leave the doctor right away, go into the pharmacy. You don't have to get on the bus and go into town.

I: Does this mean that for this decision, the first sentence, so the structures of health care, and not the shared decision-making with the doctor is important? Or do these things belong together?

P: That I decide together with the doctor...well yes, I have to know that. They say too little, mostly they don't involve us so much. They have to talk more with us, explain more, explain more!

I: Okay, that means you say for you...the organization of the health [care] system, if you could wish for something, you would like the doctor, physiotherapy, pharmacy to be all under one roof....

P: Yes, if it's all like in the medical center....

I: And on top of that, [you] would also like the doctor to include [you] and that's a good health care system for [you]?

P: Yes...yes.

I: And you're not saying that the structure is important, but the doctor should decide alone? So you're not saying I want a medical center but the doctor should decide alone without me?

P: No so he has to decide with me, he also has to explain it to me in detail. [P elaborates on her own experience on lack of shared decision-making with a doctor] So, I haven't heard anything, they write [me] and say "come and see us again" - yes, but I didn't hear that [when I talked to the doctor].

I: Okay, so you think that [centralized / integrated structures + shared decision making] belongs together and you think it's good how that is presented [in the survey].

P: Yes exactly. I would like him to explain [things] to me in detail. I would also like to see [the image diagnostics] - I haven't seen anything yet. Earlier in the GDR times, they [showed] the X-ray […] and so on.

I: Okay, yes, and in the GDR you had everything under one roof in the polyclinics, so as you said - I have the pharmacy, I have the doctor - you think that's good, don't you?

P: Yes, and they also talked with us more. Supposedly today they don't have time, there are too many people. But I think there were [more patients] in the past. I don't know.
I: Okay, but it's good that you say that, because we are still discussing a bit whether it [the descriptions for the sub-criteria related to criterion 6] was too much. But people just keep saying, like you, that [centralized / integrated structures + shared decision making] belongs together, that was also the case back then, in the GDR.
P: Yes, and that was good! There were many things that were good, they should have adopted them all!
